# *Drosophila Ubiquitin C-Terminal Hydrolase* Knockdown Model of Parkinson’s Disease

**DOI:** 10.1038/s41598-018-22804-w

**Published:** 2018-03-13

**Authors:** Hiep H. Tran, Suong N. A. Dang, Thanh T. Nguyen, Anh M. Huynh, Linh. M. Dao, Kaeko Kamei, Masamitsu Yamaguchi, Thao T. P. Dang

**Affiliations:** 1 Department of Molecular and Environmental Biotechnology, Faculty of Biology and Biotechnology, University of Science, Vietnam National University – Ho Chi Minh City, Ho Chi Minh City, 70000 Vietnam; 2 Department of Biomolecular Engineering, Kyoto Institute of Technology, Kyoto, 606-8585 Japan; 3 Department of Applied Biology, Kyoto Institute of Technology, Kyoto, 606-8585 Japan; 4 The Center for Advanced Insect Research Promotion, Kyoto Institute of Technology, Kyoto, 606-8585 Japan

## Abstract

Parkinson’s disease (PD) is the second most common neurodegenerative disorder worldwide. Many factors have been shown to contribute to its pathogenesis including genetic and environmental factors. *Ubiquitin C-terminal hydrolase L1* (*UCHL1*) is also known to be involved in the pathogenesis of PD. We herein modeled the study of *UCHL1* in *Drosophila melanogaster* and investigated its functions in PD. The specific knockdown of the *Drosophila* ortholog of *UCHL1* (*dUCH*) in dopaminergic neurons (DA neurons) led to the underdevelopment and/or degeneration of these neurons, specifically in DL1 DA neuron cluster in the larval brain lobe and PPM2, PPM3, PPL2ab, and VUM DA neuron clusters in the adult brain. These defects were followed by a shortage of dopamine in the brain, which subsequently resulted in locomotor dysfunction. The degeneration of DA neurons in *dUCH* knockdown adult brain, which occurred progressively and severely during the course of aging, mimics the epidemiology of PD. DA neuron and locomotor defects were rescued when *dUCH* knockdown flies were treated with vitamin C, a well-known antioxidant. These results suggest that *dUCH* knockdown *fly* is a promising model for studying the pathogenesis and epidemiology of PD as well as the screening of potential antioxidants for PD therapeutics.

## Introduction

Parkinson’s disease (PD) is one of the most common types of neurodegenerative disorders worldwide and is characterized by impaired locomotive ability. Mutations or alterations in single genes such as *α-synuclein*, *LRRK2*, *parkin*, *DJ-1*, and *PINK1* have been implicated in its pathogenesis. However, recent studies on PD proposed that the interaction of genetic and environmental factors including aging plays a vital role in its pathogenesis^1^ and its prevalence increases with aging^2,3^.

Ubiquitin C-terminal hydrolase L1 (UCHL1) protein has been detected in the Lewy bodies of nerve cells in PD brains^4^. The first mutation in UCHL1 (*UCHL1*^*I93M*^) was identified in two siblings of a PD family^5,6^. In contrast, *UCHL1*^*S18Y*^ mutation was shown to reduce the risk of developing PD due to its specific antioxidant protective function^7–10^. However, epidemiological studies demonstrated that UCHL1^S18Y^ mutation did not exhibit any protective functions against PD^11,12^. Another mutation in UCHL1 (*UCHL1*^*E7A*^) has been associated with progressive visual loss due to optic atrophy, but without PD symptoms^13^. Studies on UCHL1 in mouse models (gracile axonal dystrophy, *UCHL1* knockout, and *nm3419* mice) showed that the lack of UCHL1 resulted in motor ataxia, the degeneration of axons, and monoubiquitin instability^14–16^. Transgenic mice expressing UCHL1^I93M^ mutant form exhibited dopaminergic neuron (DA) degeneration under MPTP-treated conditions^17^, while the expression of UCHL1^S18Y^ mutant form exerted protective effects against MPTP toxicity^18^.

In PD research, *Drosophila melanogaster* has served as a valuable model to obtain insights into the important features of its pathogenesis^19–22^. In *Drosophila*, most DA neurons in the larval central brain are generated at embryogenesis, then mature and gather into clusters in early first instar larva^23^. However, there are also new neurons that may be generated from neuroblasts and/or existing DA neurons. These neurons may be remodeled during metamorphosis to form the mature and integral DA neuron system in the adult brain^24,25^. *Drosophila* DA neurons in larval and adult brains are divided into clusters based on the cell bodies and dendrite projections of these neurons^24^. In the third instar larval (L3) central brain, seven clusters of DA neurons have been named according the position of the cell body within the brain hemisphere, namely, DM (dorsal medial) and DL (dorsal lateral): DM1a, DM1b, DM2, DL1a, DL1b, DL2a, and DL2b^23^. In the central brain of adult fly, the distribution of DA neurons has been categorized in detail, and these neurons have been classified into nine distinct DA neuron clusters: PAM, PAL, PPM1, PPM2, PPM3, PPL1, PPL2ab, PPL2c, and VUM, which may be distinctively recognized by the position of the cell body, dendrites, and number of DA neurons in each cluster^26,27^.

Although mouse models are employed to study the functions of *UCHL1*, a *Drosophila* model is still needed to track the integrity of the whole DA neuron system, analyze neurodegeneration with a large number of animals in order to study PD at the population level, and for high-throughput genetic and drug screening. Therefore, we herein attempted to establish a new *Drosophila* model for these purposes by knocking down the *Drosophila* homolog of human *UCHL1* (*dUCH*) in the DA neuron system of fly brain, and then evaluating the effects of this knockdown on PD-related symptoms including tissue morphology, locomotor behaviors, dopamine production, DA neuron integrity, and the progression of DA neuron degeneration.

## Results

### *Drosophila* UCH (dUCH) is the ortholog of human and mouse UCHL1

**Figure 1:**
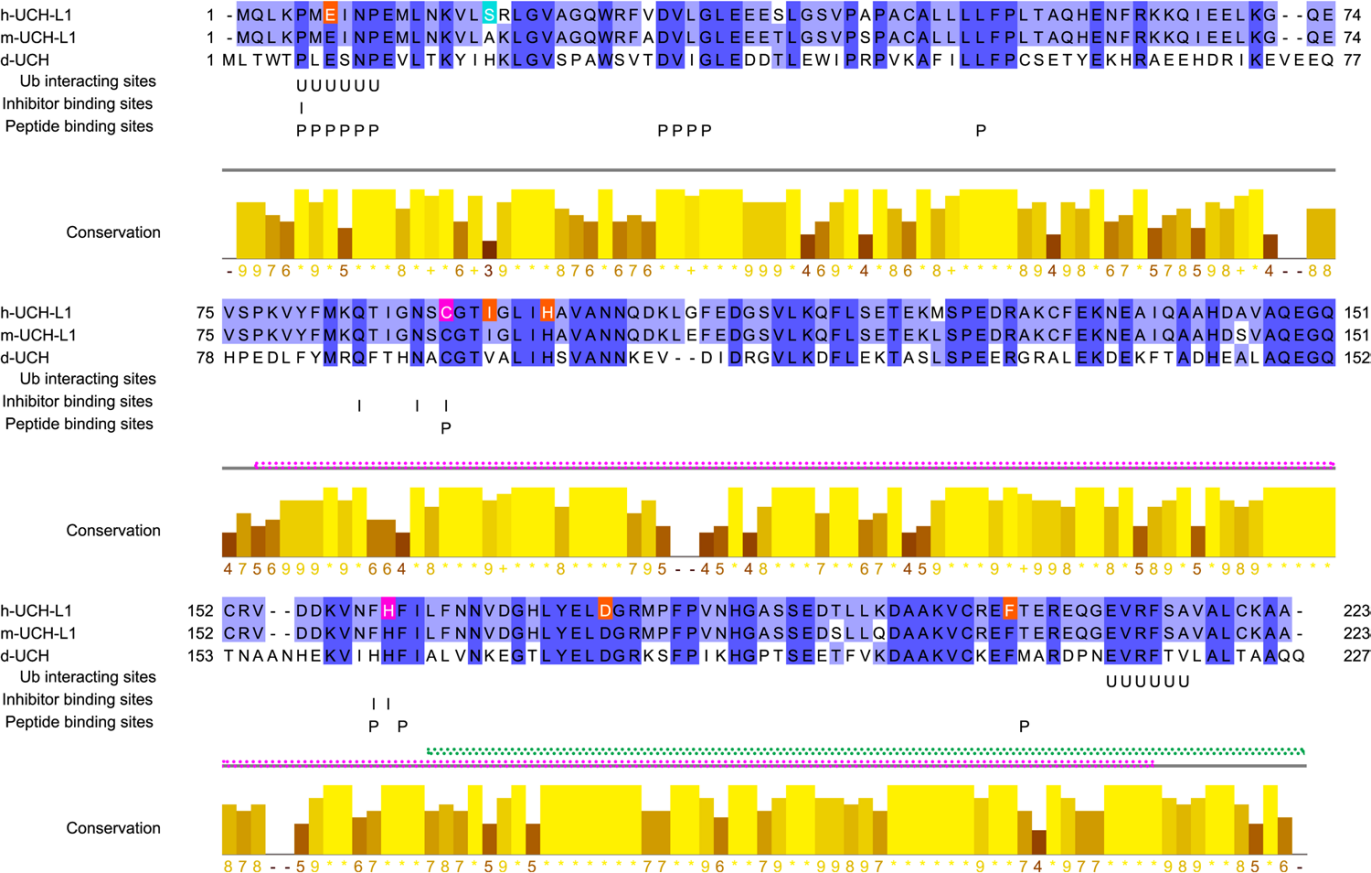
*Drosophila* ortholog of human and mouse UCHL1. hUCHL1, *Homo sapiens* UCHL1; mUCHL1, *Mus musculus* UCHL1, and dUCH, *Drosophila melanogaster* UCH. The degree of similarity is represented by the Jalview^56^ conservation score (range from 0 to 11; *score of 11, identical amino acids; + score of 10, all properties are conserved) and annotation (a higher column indicates greater conservation). Two active sites C90^29–31^ and H161^29,30^ of hUCHL1 are conserved in the mouse and fly (pink highlighted amino acids). Five important residues for hydrolytic activity: E7^13^, I93^5,64^, H97^29^, D176^29^, and F204^31^, are also conserved (orange highlighted amino acids). The turquoise highlighted amino acid, residue S18^6^, which is involved in the dimerization and ligase activity of hUCHL1, is poorly conserved. The sites for ubiquitin, inhibitor, and peptide binding (U, I, and P, respectively) are also placed on the conserved regions. Magenta and green double dotted lines show target regions for dsRNAs of VDRC *dUCH* RNAi line #26468 and #103614, respectively. Part of #103614 dsRNA bound to the 3′-UTR of *dUCH* mRNA is not shown in this figure.

In an effort to use *Drosophila melanogaster* as a model organism for characterizing UCHL1 functions in the pathogenesis of PD, we initially aligned *Drosophila* Ubiquitin C-terminal hydrolase (dUCH, P35122, CG4265) protein with human UCHL1 (hUCHL1, P09936) and mouse UCHL1 (mUCHL1, Q9R0P9). The identity of hUCHL1, mUCHL1, and dUCH was 42.7%, hUCHL1 and dUCH was 43.7%, and mUCHL1 and dUCH was 43.7%. hUCHL1, mUCHL1, and dUCH possessed long sequences (>200 residues) with high identity (>40%). Thus, these proteins appeared to share consistent structures^28^. Identical amino acids in the alignment were marked by dark and pale blue (Fig. 1). Two of the active sites (C90 and H161)^29–31^ and four important sites for hydrolytic activity (E7, H97, D176, and F204)^13,29,31^ were accommodated in identical positions and the remaining site for hydrolytic activity, I93 was placed in a high conservation position (Jalview conservation score of 9) (Fig. 1). Nevertheless, S18, which was previously suggested to be an important site for dimerization and ligation^6^, was placed in a poorly conserved position (Fig. 1). Furthermore, hUCHL1 belongs to the deubiquitinating enzyme family, which is characterized by binding to ubiquitinated proteins and cleaving peptide bonds between ubiquitin and its substrate protein (Fig. 1). Therefore, the ubiquitin-interacting sites^32^ and peptide-binding sites, which are inferred from the cysteine peptidase C12 containing Ubiquitin C-terminal hydrolase (UCH) families L1 and L3 domain conservation^33^, were added to the alignment in order to evaluate conservation (Fig. 1). The results obtained showed that all interacting and binding sites (abbreviated by the letter U and P) were placed in highly conserved regions (Fig. 1). Furthermore, most of the inhibitor binding sites (abbreviated by the letter I) were accommodated in highly conserved residues (Fig. 1). *Drosophila melanogaster* also possesses two other *UCH* genes (*CG3431* and *CG1950*); however, sequence identities indicate that these genes are not related to human *UCHL1* and *dUCH* (*CG4365*) (Supplementary Table S1). Therefore, we confirmed that dUCH is truly the ortholog of hUCHL1 and mUCHL1.

### DA neuron-specific *dUCH* knockdown flies exhibit locomotor dysfunction

**Figure 2:**
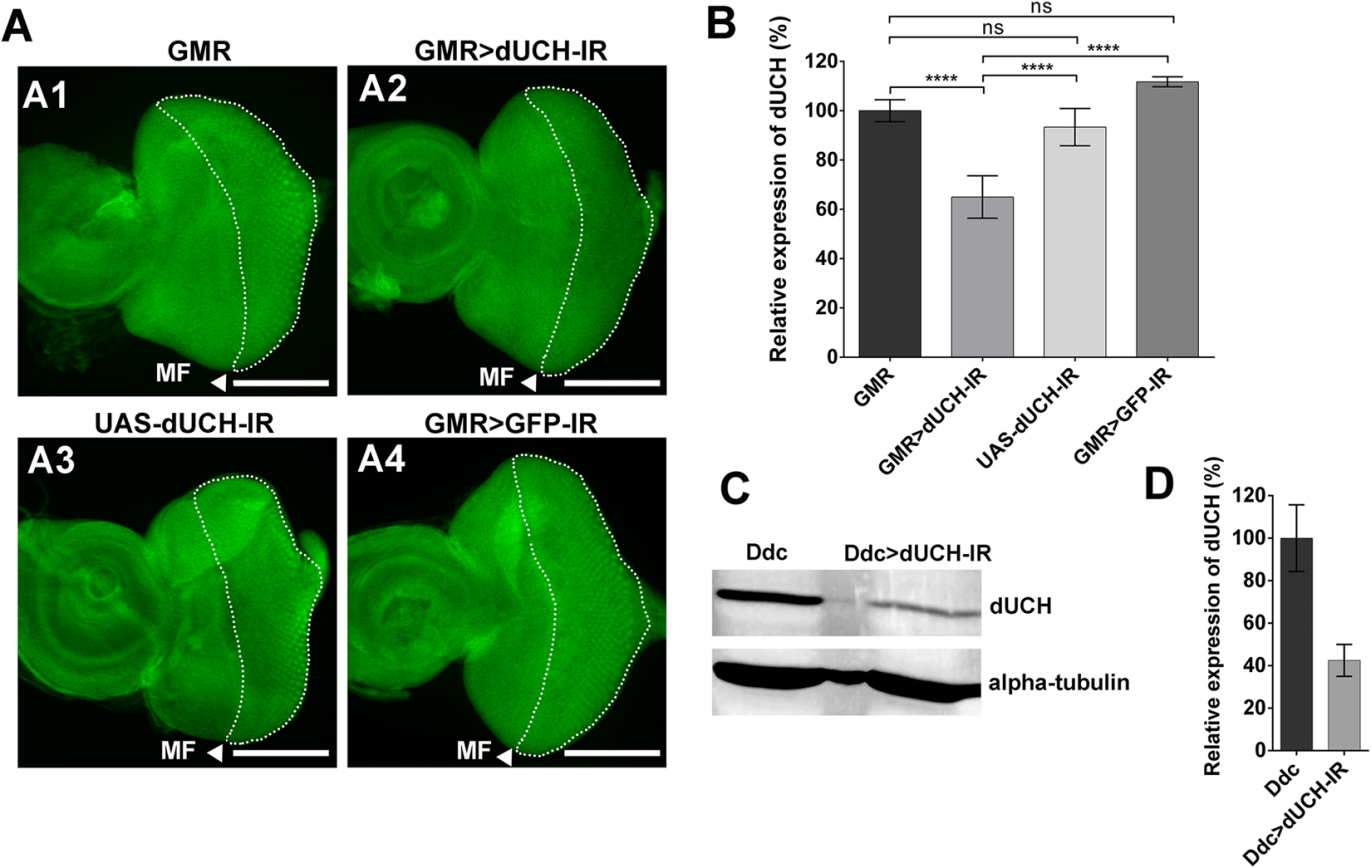
Knockdown of *dUCH* in various tissues of *Drosophila melanogaster*. (**A**) *dUCH* was knocked down in third instar larval (L3) eye imaginal discs. *GMR-GAL4* driver was activated in the eye imaginal discs from the posterior region to morphogenetic furrow (MF, white arrow head) which marked by dash line. The dUCH signal in the indicated region was weaker in the third instar larval eye imaginal discs of knockdown flies (*GMR-GAL4/*+; +; *UAS-dUCH-IR/*+, A2) than in those of driver control (*GMR-GAL4/*+; +; +, A1), *dUCH* RNAi line (+; +; *UAS-dUCH-IR/*+, A3), and dsRNA control (*GMR-GAL4/*+; *UAS-GFP-IR/*+; +, A4). Scale bars indicates 100 μm. (**B**) The quantification of dUCH relative intensity in *dUCH* knockdown (GMR > dUCH-IR), driver control (GMR), *dUCH* RNAi line (UAS-dUCH-IR), and dsRNA control (GMR > GFP-IR) eye discs. While *dUCH* RNAi line, dsRNA control showed no significant (ns) difference in dUCH intensity comparing to driver control, the dUCH signal in the knockdown discs were significant reduced comparing to all the controls. One-way ANOVA with Bonferroni’s post-hoc test, n = 5, ****p < 0.0001. (**C**) Reduction of dUCH in the adult brain. *Ddc-GAL4* drives the knockdown of *dUCH* in dopaminergic and serotonergic neurons. The intensity of the dUCH band in knockdown flies (+; +; *Ddc-GAL4/UAS-dUCH-IR*, right lane) and control flies (+; +; *Ddc-GAL4/*+, left lane) showed a reduction in dUCH in knockdown flies. (**D**) The quantification of band intensity shows the relative expression of dUCH in knockdown (Ddc) versus control (Ddc > dUCH-IR) flies. The effects of *dUCH* knockdown in various tissues were summarized in Supplementary Table S2.

We firstly examined the knockdown efficiency by using *dUCH* RNAi line (VDRC #26468, Fig. 1) under the control of GAL4-UAS system. By using the *GMR-GAL4* driver which target to the posterior region of morphogenetic furrow (MF) in eye imaginal discs, the reduction of dUCH protein levels were clearly observed (Fig. 2A and B). In the driver control (GMR), dUCH showed high expression in eye imaginal discs, especially in the posterior region of MF (Fig. 2A1). In contrast, the knockdown of *dUCH* driven by *GMR-GAL4* (GMR > dUCH-IR) clearly showed the reduction of dUCH in the posterior region (Fig. 2A2). In addition, the expression pattern of dUCH in the *dUCH* RNAi line alone (UAS-dUCH-IR, Fig. 2A3) and the control which express dsRNA of GFP (GMR > GFP-IR, Fig. 2A4) are similar with the driver control (Fig. 2A1), that may exclude the side effects of insertional transgene or expressing dsRNA in expression level of dUCH protein. Quantified intensity of dUCH signals in this area strongly confirmed the significant reduction of dUCH in *dUCH* knockdown flies compared to those of control flies (Fig. 2B). We also confirmed the knockdown efficiency of this RNAi line in brain tissues. The knockdown of *dUCH* in dopaminergic and serotonergic neurons using *Ddc-GAL4* driver (Ddc > dUCH-IR) also showed decrease in dUCH signal (Fig. 2C and D) comparing with driver control (Ddc). We then employed the knockdown of *dUCH* in a number of *Drosophila* tissues in order to examine the effects of the knockdown of *dUCH* on these tissues. The knockdown of *dUCH* was followed by the display of aberrant phenotypes (Supplementary Table S2), including locomotor dysfunction and deficits that mimic PD motor symptoms in DA neuron-specific *dUCH* knockdown flies driven by the *TH-GAL4* driver. The knockdown of *dUCH* by a different dsRNA-targeted region (VDRC #103614, Fig. 1) also showed locomotor deficits as well as reductions in the number of DA neurons (Supplementary Fig. S1). These results exclude the possible off-target effect and, thus, the observed phenotypes with these *dUCH* RNAi lines are truly caused by a reduction in dUCH. In this study, the term *dUCH* knockdown and *dUCH* RNAi line was used for VDRC #26468 line unless otherwise indicated.

**Figure 3:**
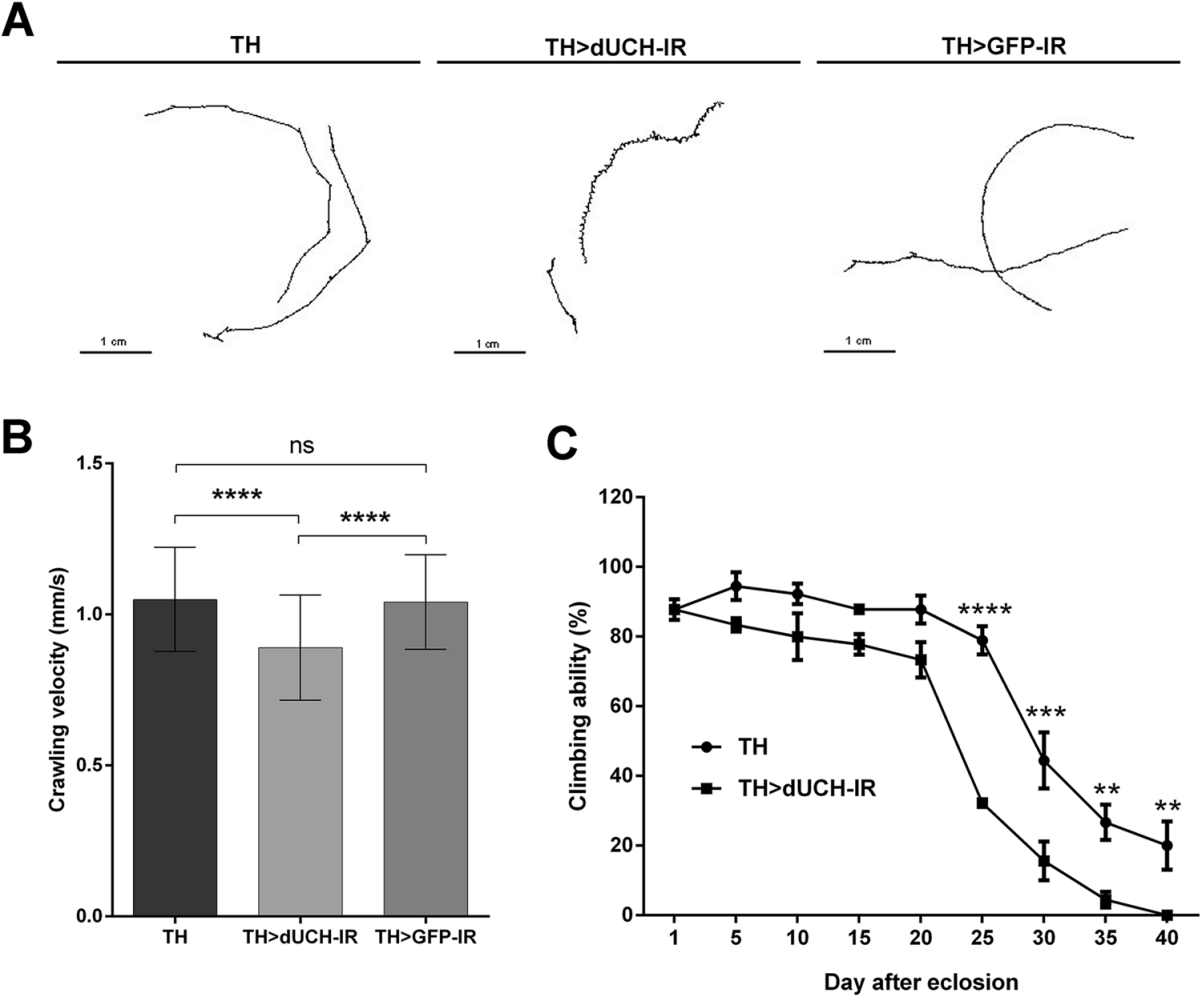
Dysfunction in the locomotor behaviors of dopaminergic neuron-specific *dUCH* knockdown flies. (**A**) Motion paths of driver control (+; +; *TH-GAL4/*+, left panel), *dUCH* knockdown (+; +; *TH-GAL4/UAS-dUCH-IR*, middle panel), and dsRNA control (+; +; *TH-GAL4/UAS-GFP-IR*, right panel). *dUCH* knockdown larvae exhibited shorter crawling paths comparing to the driver and dsRNA control larvae. (**B**) Crawling velocity of driver control (TH), *dUCH* knockdown (TH > dUCH-IR), and dsRNA control larvae (TH > GFP-IR), n = 37, one-way ANOVA with Tukey’s multiple comparisons test, ****p < 0.0001, data are presented as mean ± SD. (**C**) Climbing ability of driver control (TH) and *dUCH* knockdown adult flies (TH > dUCH-IR). Knockdown flies started to exhibit decline in climbing ability at day 25 after eclosion, as analyzed by repeated measures of two-way ANOVA with Bonferroni’s post hoc test, n = 30, **p < 0.01, ***p < 0.001, ****p < 0.0001, data are presented as the mean ± S.E.M. The consistent results in four other independent cohorts were shown in Supplementary Fig. S2. The same effect was also observed in different line of *dUCH* knockdown flies (Supplementary Fig. S1).

To further analysis the effects of *dUCH* knockdown in DA neuron-specific *dUCH* knockdown flies, we continue focused on examining the moving ability of these files, which already showed some remarkable impacts in primary screening mentioned above. The effects of *dUCH* knockdown on L3 wandering behavior were examined using crawling assay. Heterozygous *dUCH* knockdown larvae displayed a shorter moving path (Fig. 3A, middle panel) than the driver controls (Fig. 3A, left panel), and dsRNA controls (Fig. 3A, right panel) in an identical interval of time. The mean velocity of knockdown larvae was reduced comparing to both controls (Fig. 3B). The consistent results were observed in four other independent cohorts (Supplementary Fig. S2). In the adult stage, startle-induced negative geotaxis assay showed a decline in the climbing ability of heterozygous *dUCH* knockdown flies from that by the driver controls (Fig. 3C). The knockdown and control flies showed age-related declines in climbing ability (Fig. 3C). The percentage of flies that climbed across the 10-cm mark of the cylinder in 10 sec was reduced throughout consecutive time-points (Fig. 3C). This percentage was 88% in 1-day-old and 20% in 40-day-old control flies, and was 88% in 1-day-old and 0% in 40-day-old knockdown flies. At each time-point, there was no significant difference in the climbing activity between the control and knockdown flies until these flies reached 20 days old (repeated measures two-way ANOVA with Bonferroni’s post hoc test, p > 0.05). The climbing ability of knockdown flies markedly decreased at 25 days old, leading to a significant difference at this time-point (repeated measures two-way ANOVA with Bonferroni’s post hoc test, p < 0.0001). The decline observed in the climbing function of knockdown flies was sustained from 30 days old (repeated measures two-way ANOVA with Bonferroni’s post hoc test, p < 0.001 at 30 days old, p < 0.01 at 35 and 40 days old) and reached its highest level, at which no fly climbed across the 10-cm mark by 10 sec, in 40-day-old flies. Collectively, these results demonstrate that the reduction in dUCH in the *Drosophila* brain, specifically in DA neurons, led to a disorder in crawling behavior and decline in locomotor ability.

### *dUCH* knockdown larvae exhibit reductions in DL1 DA neuron numbers

**Figure 4:**
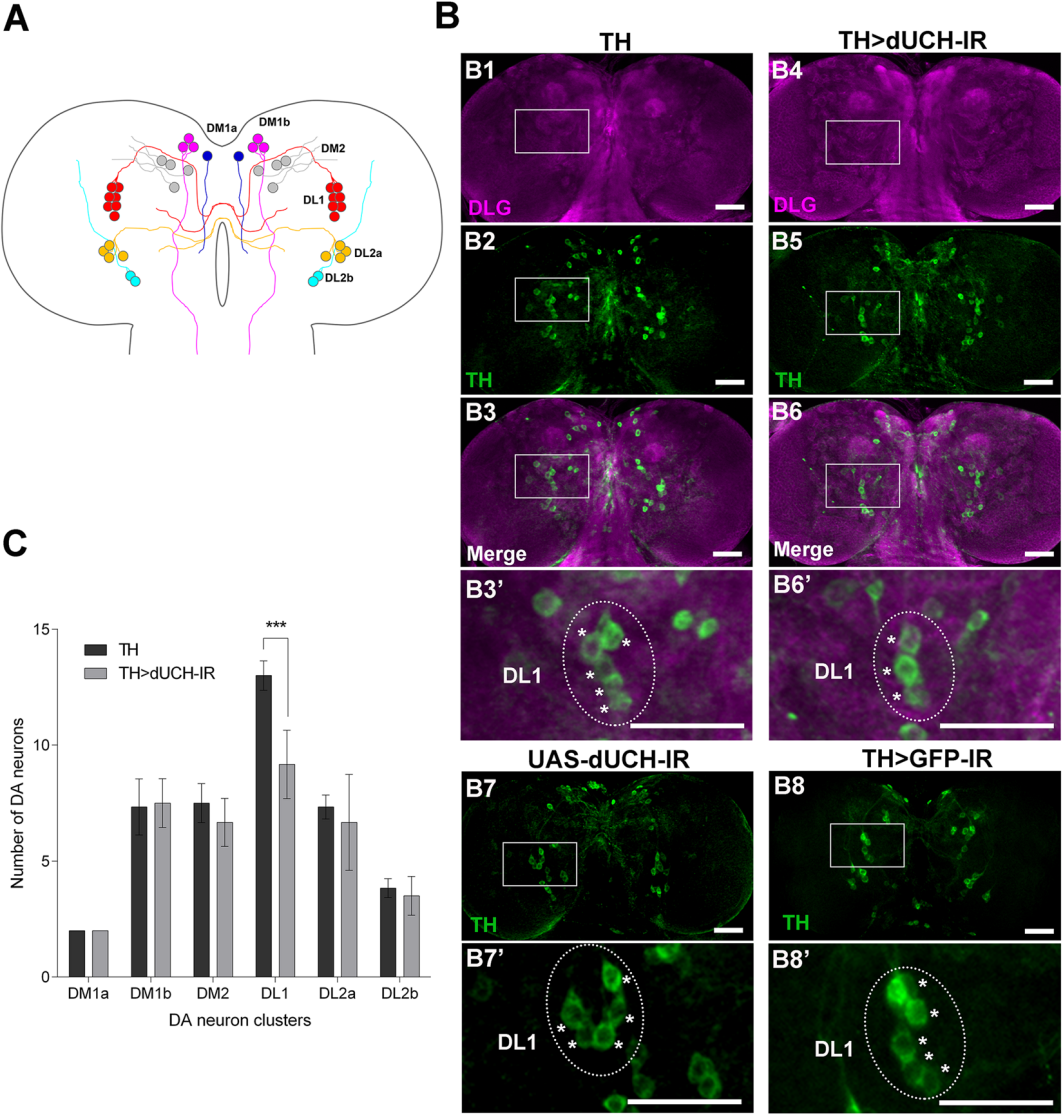
Abnormalities in the number of DL1 dopaminergic neurons in the *dUCH* knockdown larval brain. (**A**) A schematic representation of six DA neuron clusters (DM1a, DM1b, DM2, DL1, DL2a, and DL2b) and projections in *Drosophila* L3 central brain was illustrated based on previous studies^23,24^. (**B**) Representative confocal images show DA neuron clusters in the third instar larval central brain immunostained with the anti-TH antibody (TH, green). The whole brain was counterstained with anti-DLG (DLG, magenta). Driver control flies (+;+; *TH-GAL4/*+) are shown in the left panel (B1, B2, B3, B3’) and *dUCH* knockdown flies (+; +; *TH-GAL4/UAS-dUCH-IR*) in the right panel (B4, B5, B6, B6’). The boxed area in the merged image (B3, B6) marks the DL1 cluster, which is magnified in panels B3’ and B6’. The number of DA neurons in the DL1 cluster was less in the *dUCH* knockdown brain (B6’) than in the driver control brain (B3’). This effect was not detected in *dUCH* RNAi line (+; +; *UAS-dUCH-IR/*+, B7-B7’), and dsRNA control (+; +; *TH-GAL4/UAS-GFP-IR*, B8-B8’). DL1 DA neuron cluster in the boxed area of driver control, *dUCH* knockdown, *dUCH* RNAi line, and dsRNA control was respectively shown in B3’, B6’, B7’, and B8’. (**C**) Quantification of DA neurons in each cluster for both brain hemispheres in driver control flies (black bars) and *dUCH* knockdown flies (gray bars). The decrease in number of DA neurons in DL1 cluster was observed in *dUCH* knockdown brains (TH > dUCH-IR) comparing to driver control brains (TH), unpaired Student’s *t*-test with Welch’s correction, n = 6, ***p < 0.001 (data are presented as the mean ± SD). Scale bars indicate 50 μm. DA neuron, Dopaminergic Neuron; DM, Dorsal Medial; DL, Dorsal Lateral; TH, Tyrosine Hydroxylase; DLG, *Drosophila* Discs Large. The same effect was also observed in different line of *dUCH* knockdown flies (Supplementary Fig. S1).

Previous studies reported that locomotor dysfunctions in PD patients are caused by the degeneration of DA neurons^34,35^. These neurons play important roles in dopamine production in the central nervous system and control multiple functions of the brain including voluntary movement. In *Drosophila*, locomotor deficits were observed with the ectopic expression of various PD-related genes such as *α-synuclein*, *parkin*^*R275W*^, and *LRRK2*^19,20,36^, and were accompanied by the degeneration of DA neurons. In the present study, we initially examined the number of DA neurons in larval *dUCH* knockdown brains using immunoreactivity to the rate-limiting enzyme, tyrosine hydroxylase (TH). In the L3 central brain, the pattern, shape, and number of DA neurons in most of the clusters of *dUCH* knockdown flies (TH > dUCH-IR, Fig. 4-B4-B6’) were not significantly different from those of driver control flies (TH, Fig. 4-B1-B3’), except for the DL1 cluster (Fig. 4-B6’ and 4-B3’). Neither *dUCH* RNAi line (UAS-dUCH-IR) nor dsRNA control (TH > GFP-IR) showed defect in DL1 cluster (Fig. 4-B7,B7’ and 4-B8,B8’). The DL1 cluster was the only cluster that showed a significant reduction in the number of DA neurons between *dUCH* knockdown and driver control flies (Fig. 4C). In addition, knockdown of *dUCH* by different target region of dsRNA also showed similar effect in decrease of locomotor ability and degeneration of DA neuron (Supplementary Fig. S1). These results indicate that a reduction in dUCH may lead to the incomplete loss or underdevelopment of DA neurons in the DL1 clusters of L3 central brains.

### **DA neurons in*dUCH*** knockdown adult brains progressively degenerated **with aging**

**Figure 5:**
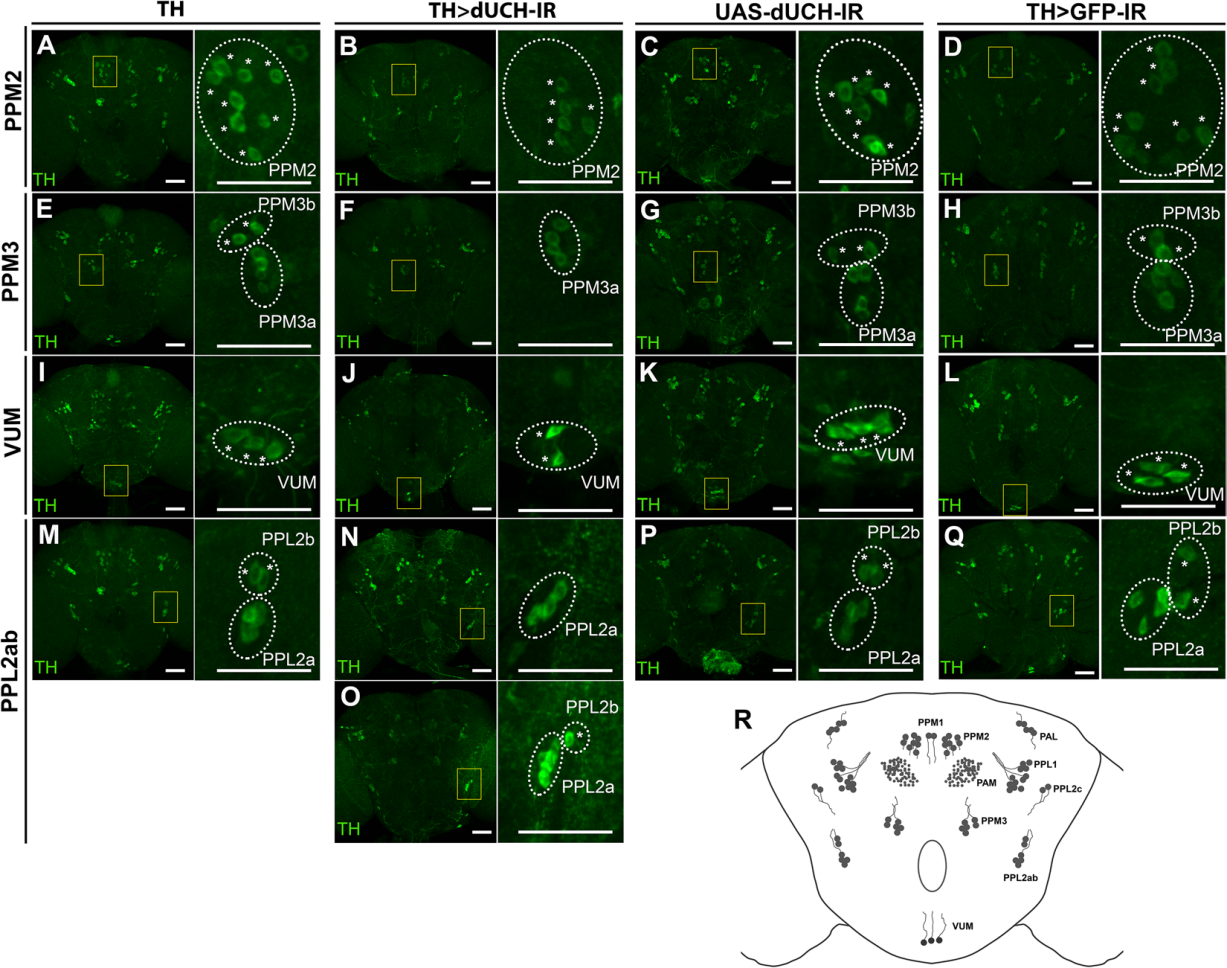
The degeneration of DA neurons in the *dUCH* knockdown adult brain. (**A–Q**) Confocal images represent four DA neuron clusters PPM2 (**A–D**), PPM3 (**E–H**), VUM (**I–L**), and PPL2ab (**M–Q**) in the adult brains of driver control (TH), *dUCH* knockdown (TH > dUCH-IR), *dUCH* RNAi line (UAS-dUCH-IR), and dsRNA control (TH > GFP-IR) flies. DA neurons were stained with the anti-TH antibody. The boxed areas capture DA neuron clusters that are magnified on the right side of each panel. Asterisks were used to mark individual DA neurons in indicated clusters. *dUCH* knockdown brains showed DA neuron degeneration in PPM2, PPM3, VUM, and PPL2ab comparing to driver control and the degeneration was not observed in *dUCH* RNAi line and dsRNA control. The degeneration of DA neurons in these clusters was confirmed by capturing DA neurons with both anti-TH and nuclear GFP signals (Supplementary Fig. S3). PPM2 and VUM showed the loss of one or some DA neurons (**B** and **J**), while PPM3 and PPL2ab showed losses at specific DA neuron sub-clusters (two neurons in the PPM3 sub-cluster; one or two neurons in PPL2b) (**F**,**N**,**O**). Scale bars indicate 50 µm. (**R**) The schematic representation of DA neuron clusters in the *Drosophila* adult brain was illustrated based on previous studies^26,27,37^.

In the *Drosophila* adult brain, DA neurons have been classified into nine clusters (PAM, PAL, PPM1, PPM2, PPM3, PPL1, PPL2ab, PPL2c, and VUM) characterized by cell body position and dendrite projections, as described previously^26,27,37^ (Fig. 5R). While the adult brains of driver control (TH), *dUCH* RNAi line (UAS-dUCH-IR), and dsRNA control (TH > GFP-IR) showed no evidence of DA neuron degeneration, *dUCH* knockdown (TH > dUCH-IR) exhibited the prominent loss of DA neurons in PPM2, PPM3, PPL2ab, and VUM clusters (Fig. 5). In PPM2 and VUM, one or some DA neurons in *dUCH* knockdown brain had degenerated relative to those control flies, whereas others still remained (PPM2, Fig. 5B comparing to controls A, C, and D; VUM, Fig. 5J comparing to controls I, K, and L). These results may be explained by the random loss of DA neurons based on differences in the susceptibility of neurons to the lack of dUCH conditions. In PPM3 cluster of *dUCH* knockdown brain, the complete loss of DA neurons occurred in a two-neuron sub-cluster of PPM3 (PPM3b), whereas the other four-neuron sub-cluster (PPM3a) maintained its integrity (Fig. 5F comparing to controls E, G, and H). These results indicate that the lack of dUCH also leads to the selective loss of DA neurons at the sub-cluster level. PPL2ab showed the characteristics of random and selective loss, exhibiting either the complete loss of two neurons in PPL2b (Fig. 5N comparing to controls M, P, Q) or the random loss in PPL2b with the degeneration of one DA neuron (Fig. 5O comparing to control M, P, Q). In order to confirm whether the loss of anti-TH signals correlated with loss of DA neurons or somehow by the decrease expression level of TH, we generated DA-neuron-specific *dUCH* knockdown flies in which DA neurons marked with nuclear GFP. Immunostaining the brain of these flies with anti-TH antibody showed the co-localization of anti-TH signals and nuclear GFP signals in almost DA neurons observed (Supplementary Fig. S3). Moreover, the simultaneous loss of both anti-TH and nuclear GFP signals in the four examined clusters were also noticed and this is in consistent with previous data. These observations strongly confirmed the degeneration of these DA neurons in *dUCH* knockdown flies. Taken together, these results demonstrated that lack of dUCH led to degeneration of DA neurons detected in four clusters of adult brain.

**Figure 6:**
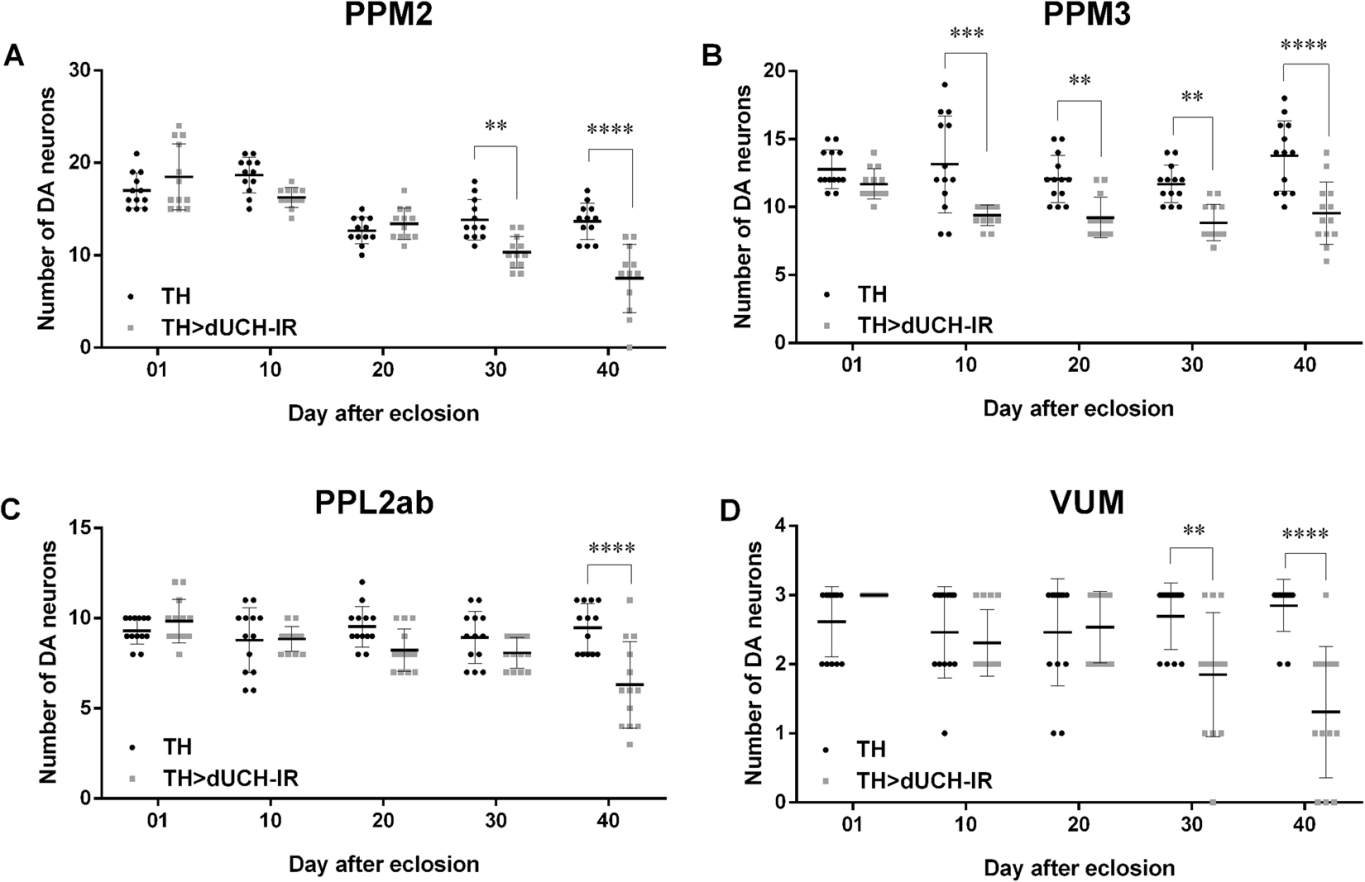
Age-related neuronal vulnerability and progressive loss of DA neurons in *dUCH* knockdown adult flies. The number of DA neurons per brain from day 1 to 40 after eclosion in PPM2 (**A**), PPM3 (**B**), PPL2ab (**C**), and VUM (**D**) clusters of driver control (TH) and *dUCH* knockdown brains (TH > dUCH-IR). *dUCH* knockdown adult brains showed significant reduction of DA neurons differently among clusters and the reduction became severer with aging; ordinary two-way ANOVA following by Sidak’s post hoc test; n = 12 adult flies (PPM2) and n = 13 adult flies (PPM3, PPL2ab, VUM); **p < 0.01, ***p < 0.001, ****p < 0.0001. Data are presented as the mean ± SD.

PD is characterized by the progressive loss of DA neurons with aging. Hence, we quantified the numbers of DA neurons in PPM2, PPM3, PPL2ab, and VUM from 1- to 40-day-old fly brains at 10-day intervals. The results obtained showed that *dUCH* knockdown brains had significantly lower numbers of DA neurons in PPM2, PPM3, PPL2ab, and VUM than in control flies at 40 days old (Fig. 6A–D). However, the age at which *dUCH* knockdown flies exhibited the significant degeneration of DA neurons varied from cluster to cluster. The reduction of DA neurons was first observed in PPM3 at 10 days old (Fig. 6B). PPM2 and VUM showed a significant loss starting at 30 days old (Fig. 6A and D), whereas degeneration in PPL2ab started at 40 days old (Fig. 6C). These results indicated that the degeneration of DA neurons in *dUCH* knockdown brains did not occur immediately at a certain time point but proceeded gradually at different time points with aging. Degeneration began in PPM3, followed by PPM2 and VUM, and the most severe degeneration occurred in all four clusters, including PPL2ab, in the oldest flies in the population examined (40 days old). This result implied a difference in the susceptibility of DA neuron clusters when individual flies exhibited the lack of dUCH with aging.

### *dUCH* knockdown adult flies show a significant reduction in dopamine in the brain

**Figure 7:**
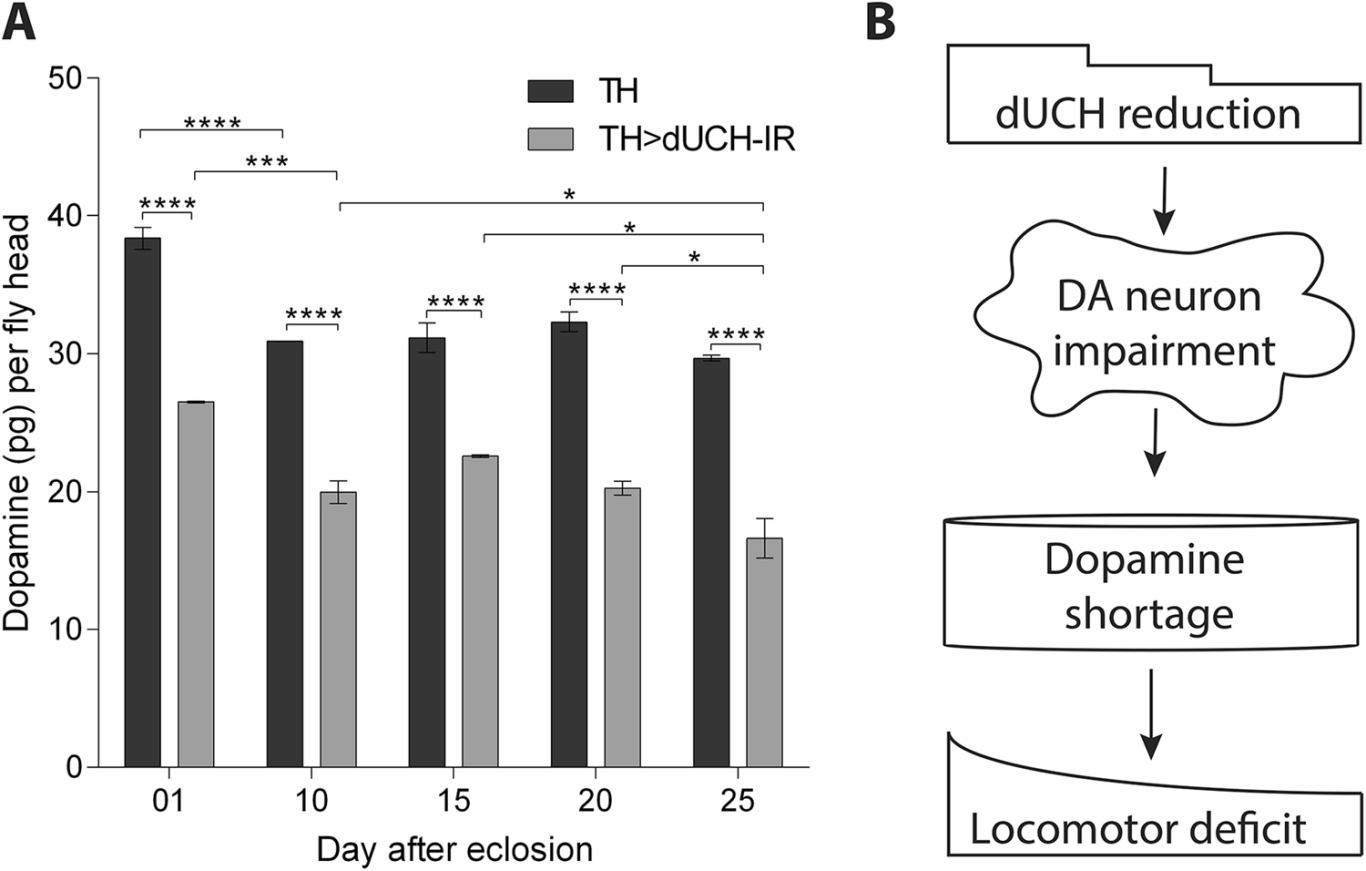
Dopamine shortage in the brain of *dUCH* knockdown adult flies. (**A**) The level of dopamine (pg) per fly head in *dUCH* knockdown flies (+; +; *TH-GAL4/UAS-dUCH-IR*) and driver control flies (+; +; *TH-GAL4/*+), two-way ANOVA with Tukey’s multiple comparisons test (n = 2 cohorts/ each genotype, see Materials and Methods), *p < 0.05, ***p < 0.001, ****p < 0.0001. Data are presented as the mean ± SD. (**B**) Graphical summary: the intermediate role of dopamine in the process from dUCH reductions to DA neuron impairment and then a locomotor deficit.

The reduction in dopamine, which is observed in the brains of PD patients, may be directly involved in PD symptoms. The production of dopamine mainly occurs in DA neurons via the catecholamine biosynthesis pathway^27,38^. In order to examine the effects of DA neuron impairments on dopamine levels in the brains of *dUCH* knockdown flies, we quantified dopamine levels in *dUCH* knockdown brains. The results obtained showed that dopamine levels were lower at every day of the examination (1, 10, 15, 20, and 25 days after eclosion) in *dUCH* knockdown flies than in driver control flies (Fig. 7A). In the period from 1 to 10 days old, *dUCH* knockdown and control flies exhibited significant reductions in dopamine levels (Fig. 7A), with fold differences of 19.5% and 24.7%, respectively. While control flies did not show any significant differences in dopamine levels from the period of 10 to 25 days old with a fold difference of 8.1% (20 versus 25 days old), knockdown flies exhibited significant reductions in dopamine levels from 10-, 15-, and 20-day-old flies to 25-day-old flies (Fig. 7A), with a fold difference of 18% (20 versus 25 days old). This contributed to the high fold difference observed between 1- and 25-day-old in *dUCH* knockdown flies of 37.3% to 22.7% in driver control flies. These results are consistent with our previous findings on climbing ability and DA neuron integrity. The significant reduction in climbing ability began in 25-day-old flies (Fig. 3C), and most DA neuron clusters (PPM2, PPM3, and VUM) exhibited degeneration in 20- to 30-day-old flies (Fig. 6A,B and D). These events perfectly matched the marked reduction observed in dopamine levels in 25-day-old *dUCH* knockdown flies with a fold difference of 18% to 8.1% in driver control flies. The reduction in dopamine in *dUCH* knockdown flies suggested a relationship between DA neuron impairments by *dUCH* knockdown and locomotor deficits. These results may be modeled as the reduction in dUCH causing impairments in DA neurons, which result in a reduction in dopamine levels followed by a dysfunction in locomotor behaviors (Fig. 7B).

### Vitamin C rescued DA neuron defects and locomotor dysfunction caused by *dUCH* knockdown

Oxidative stress has been proposed as a common factor in progressive neurodegeneration. Therefore, the protection of brain tissue against oxidative damage is one of the potential strategies to reduce the risk of developing neurodegenerative disease. Vitamin C is a key antioxidant in the central nervous system and protects the brain against neurodegenerative disorders, including Amyotrophic lateral sclerosis, Alzheimer’s disease, Huntington’s disease, and PD^39^. The treatment of PD fly models with vitamin C decreased oxidative stress^40^ and delayed the loss of climbing ability^41^, suggesting that early antioxidant supplementation is an effective therapy against neurodegenerative processes.

**Figure 8:**
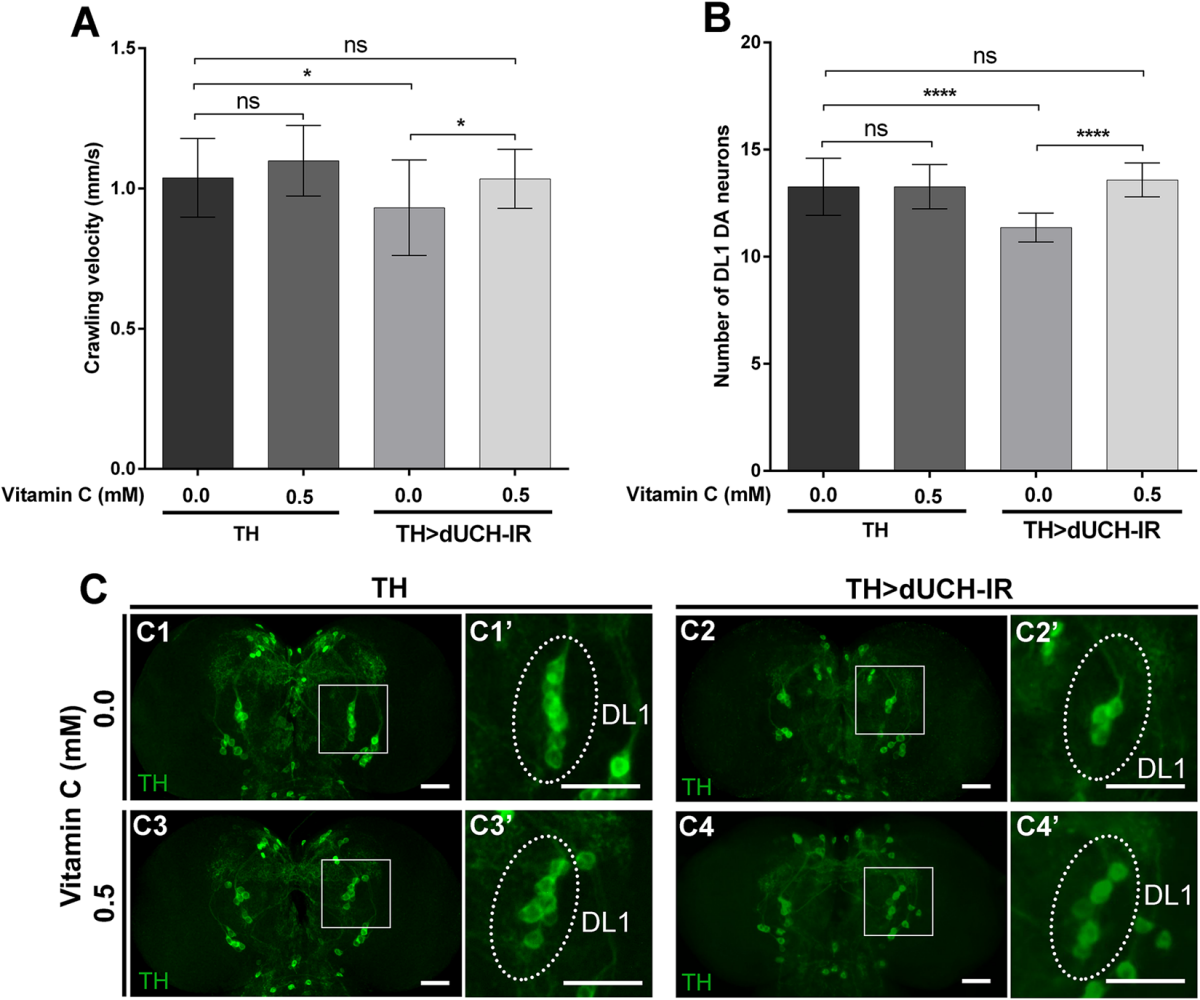
Vitamin C treatment reduced the effects of the knockdown of *dUCH*. (**A**) Crawling velocity of control (+; +; *TH-GAL4/*+) and *dUCH* knockdown larvae (+; +; *TH-GAL4/UAS-dUCH-IR*) under non-treated and treated conditions with 0.5 mM vitamin C. After being treated with vitamin C, the crawling ability of *dUCH* knockdown larvae was better than that of non-treated knockdown larvae and became similar to that of driver control larvae, the Kruskal-Wallis test following by Dunn’s post hoc test, n = 29 larvae/each genotype, NS: not significant, *p < 0.05, data are presented as the mean ± SD. (**B**) Quantification of DA neurons in the DL1 cluster of driver control and *dUCH* knockdown brain lobes. The number of DA neurons in the DL1 cluster of *dUCH* knockdown larvae treated with 0.5 mM vitamin C markedly increased, while the non-treated *dUCH* knockdown brain lobe showed the loss of DA neurons, an ordinary one-way ANOVA following by Sidak’s post hoc test; n = 11 larvae/ each genotype, NS: not significant, ****p < 0.0001, data are presented as the mean ± SD. (**C**) Representative confocal images show DA neuron clusters in the third instar larval central brain immunostained with the anti-TH antibody (TH) of the driver control (C1, C3) and *dUCH* knockdown flies (C2, C4) not treated or treated with 0.5 mM vitamin C, respectively. (C1’, C2’, C3’, C4’) Magnified pictures showed DL1 clusters demonstrating the loss of DA neurons under the effect of the *dUCH* knockdown (C2’) from that in the driver control (C1’). After the vitamin C treatment, the number of DA neurons in the DL1 cluster of the *dUCH* knockdown brain lobe (C4’) was similar to that of the driver control (C1’, C3’). Scale bars indicate 50 µm.

Since UCHL1 has been suggested as a neuronal antioxidant^42^, we investigated whether an antioxidant may reverse the effects of *dUCH* knockdown in the *Drosophila* model. As expected, our results clearly demonstrated that vitamin C at a final concentration of 0.5 mM restored the loss of DA neurons in the DL1 cluster in *dUCH* knockdown larvae (Fig. 8B,C1-C4,C1’-C4’). Consequently, the locomotor dysfunction in larvae induced by *dUCH* knockdown was also rescued when larvae were treated with 0.5 mM vitamin C (Fig. 8A). These results showed that vitamin C may prevent the underdevelopment and/or degeneration of DA neurons due to the inhibition of oxidative stress, and strongly suggest thatthe knockdown of *dUCH* is involved in defects in oxidative stress responses. Collectively, these results are consistent with the proposal that UCHL1 plays a role as an antioxidant in PD. The knockdown of *dUCH* has been confirmed for use as a valuable model to test and identify new drugs with therapeutic potential for PD.

## Discussion

*Drosophila melanogaster* possesses a conserved DA synthetic pathway and distinct DA neuron clusters^27^, and has been utilized as a model for studying many PD-related genes such as *α-synuclein*^19^, *LRRK2*, *parkin*, *DJ-1*, and *PINK*^22^. Therefore, we utilized *dUCH* knockdown flies to study the function of *UCHL1* in PD. The ubiquitous knockdown of *dUCH* led to pupal lethality. The tissue-specific knockdown of this protein resulted in phenotypic abnormalities in the eye, wing, and thorax, and particularly in locomotor behaviors. These results demonstrate that *dUCH* plays essential roles in the development as well as maintenance of the normal functions of living organisms.

Previous studies on PD-related genes using a *Drosophila* model also showed locomotor dysfunctions including declines in the climbing ability of flies overexpressing *α-synuclein*^19^ or reductions in the crawling ability of *parkin* mutant larvae^43,44^. In the present study, *dUCH* knockdown flies exhibited locomotor abnormalities in the larval and adult stages. *dUCH* knockdown L3 larvae exhibited a decline in crawling ability that appeared to be a consequence of the lack of DL1 DA neurons in the larval central nervous system. These results are similar to previous findings obtained from *PINK1* knockdown zebrafish larvae in which only one group of DA neurons decreased in number, resulting in weaker or no responses to a tactile stimulation and hypoactivity^25^. In *Drosophila*, the biogenesis of neurons in the larval brain originates from neuroblasts going through two phases during embryogenesis and during late first to second instar larvae^23^. Thus, the knockdown of *dUCH* may lead to a developmental delay or the loss of DL1 DA neurons. This result is consistent with previous findings on *UCHL1* transgenic zebrafish that demonstrated that *UCHL1* plays important roles in neuronal development in the early stage of embryos^45^.

In the adult stage, a progressive decline in climbing ability occurred in control and knockdown flies during the course of aging, and this was due to the natural aging process including declines in physiological brain functions and the degeneration of nerve cells or musculoskeletal weakness^27^. However, it is important to note that the significant reduction observed in climbing ability occurred in 25-day-old knockdown *dUCH* flies and was sustained until 40 days old. The locomotor dysfunction was also shown to be a result of DA neuron degeneration driven by mutations in some PD-related genes such as *parkin* and *LRRK2*. Previous studies on the *Drosophila* PD model showed that a mutation in *parkin* or the overexpression of *parkin* mutant gene caused the degeneration of DA neurons, specifically in the PPL1 cluster^20,26^. The overexpression of *LRRK2* wild type or its mutant led to the degeneration of DA neurons in six clusters (PAL, PPM1, PPM2, PPM3, PPL1, and PPL2)^20^. In the present study, the knockdown of *dUCH* induced the degeneration of four specific DA neuron clusters (PPM2, PPM3, PPL2ab, and VUM) out of the eight examined clusters (PAL, PPM1, PPM2, PPM3, PPL1, PPL2ab, PPL2c, and VUM) in adult brain (Fig. 5). The degeneration of DA neurons in the four clusters occurred either randomly, selectively, or in a mixed style (random and selective loss taking place in one cluster). Some PPM2 DA neurons were lost randomly, and this may have been due to the differences in the susceptibility of each individual neuron under *dUCH* knockdown conditions (Fig. 5B). In PPM3, degeneration occurred in two specific DA neurons, while the others still existed (Fig. 5F). These results indicate that DA neurons in PPM3 belong to two separated groups, which are named PPM3b sub-cluster with two specific DA neurons and PPM3a sub-cluster with the remaining four DA neurons. VUM also exhibited selective loss at one specific neuron out of the three DA neurons in the cluster (Fig. 5J). The two PPL2b neurons in PPL2ab cluster may be completely or partially lost, exhibiting the mixed style of random and selective loss (Fig. 5N and O). These results suggest that DA neuronal vulnerability under *dUCH* knockdown conditions strongly depends on clusters or sub-clusters, and individual neurons.

We monitored the DA neurons of control and *dUCH* knockdown flies throughout adulthood in 10-day intervals. The initial days of degeneration differed among the four clusters (PPM2, PPM3, PPL2ab, and VUM) (Fig. 6). The degeneration of PPM3 neurons occurred very early at day 10 post-eclosion, while the degeneration of PPL2ab started late at day 40 post-eclosion. The other clusters, PPM2 and VUM, exhibited degeneration at day 30 post-eclosion. From another point of view, at 10 and 20 days old, degeneration only occurred in PPM3. At 30 days old, PPM2, PPM3, and VUM degenerated, while all the four clusters exhibited degeneration at 40 days old. In comparison with locomotor behavior data, the decline observed in the climbing ability of *dUCH* knockdown flies started at day 25 post-eclosion, which approximately corresponded to the loss of PPM2, PPM3, and VUM (day 20 to 30 post-eclosion). These results suggest that the deficit in climbing ability is a consequence of DA neuron degeneration. The quantification of dopamine in the adult fly head showed that its levels in the knockdown fly brain were always lower than those in the control fly brain starting at day 1 post-eclosion (Fig. 7A). During *Drosophila* development, the expression of TH begins in the early first instar larval brain^23^, while that of *dUCH* begins in the early stages of embryogenesis^46^. We herein utilized the *TH-GAL4* driver to knockdown *dUCH*; therefore, the observed effects may be long lasting throughout the larval, pupal, and adult stages, resulting in a chronic deficit in dopamine in adult brains. Dopamine levels in knockdown flies rapidly decreased between 20 and 25 days old, this is consistent with previous findings obtained from α-synuclein^A53T^-overexpressing showing a reduction in dopamine at week 3 post-eclosion^47^. This period coincided with the time that PPM2, PPM3, and VUM degenerated (day 20 to 30 post-eclosion) and with the initial day of the decline in climbing ability (day 20 to 25 post-eclosion). Collectively, these results consolidate our hypothesis that the degeneration of DA neurons under *dUCH* knockdown conditions leads to the reduction of dopamine levels and results in the deficit of locomotor function. This process mimics important symptoms and pathogenic events in PD patients, demonstrating that *dUCH* knockdown fly is a potential model for studying the pathogenesis of PD.

The prevalence of PD increased with aging in the population of *dUCH* knockdown flies from an epidemiological point of view, whereas progression rates depended on the clusters (Fig. 6). This result suggests that the differences in the susceptibility of DA neurons with aging and *dUCH* knockdown conditions are responsible for the progression of PD-related symptoms in *dUCH* knockdown flies. In addition, during their lifetime, the number of *dUCH* knockdown flies with an intact DA neuron system or with the degeneration of one or two DA neuron clusters decreased, while the number of flies with the degeneration of three or four clusters increased (Fig. 6). These results indicate that when *dUCH* knockdown flies age, the risk of severe DA neuronal damage increases, clearly demonstrating that *dUCH* knockdown flies exhibit the epidemiological characteristics of PD. These results also suggest that *dUCH* knockdown fly is not only a suitable model for studying the pathogenesis of PD but also a promising model for investigating its epidemiology.

Although previous studies revealed that oxidative stress contributes to the pathogenesis of PD^48,49^, the treatment of PD patients using antioxidant supplements is challenging. For example, limited cases have successfully used vitamin C to improve PD symptoms^50^. The reason for this may be the different pathological mechanisms leading to oxidative stress in sporadic PD^51^ and natural antioxidants using different biochemical mechanisms reacting with free radicals^52^. One antioxidant that has been successfully used in one PD patient may not be applicable to others. Therefore, the screening of a number of new antioxidants as well as combinations of these antioxidants may contribute to improvements in the application of antioxidant supplements to PD treatment. In the present study, vitamin C was selected as the first step to examine the potentiality of using *dUCH* knockdown model to screen for new antioxidants and combinations for PD supplements. Vitamin C, a well-known antioxidant, rescued the climbing disability of the PD fly model expressing α-*synuclein*^41^ and suppressed the phenotype of the fly carrying *DJ-1* mutation^40^. However, UCHL1 has been suggested to play a role in oxidative stress^42^. In the present study, the loss of DA neurons in our *dUCH* knockdown flies was rescued by a treatment with 0.5 mM vitamin C, and this was followed by a significant recovery of locomotor ability. Our results suggest that *dUCH* knockdown fly has potential as a promising model for screening antioxidant candidates and combinations used as supplements for PD.

## Methods

### Fly Strains and maintenance

Fly stocks were cultured on standard food medium containing 5% dry yeast, 5% sucrose, and 1% agar under a 12-h light/dark cycle at 25 °C. When performing the assay with vitamin C, vitamin C was added to standard food at a final concentration of 0.5 mM and maintained in the dark to protect against decomposition. Wild-type strain Canton-S was obtained from the Bloomington *Drosophila* Stock Center (BDSC). *UAS-GFP.nls* stock was obtained from Kyoto Stock Center (DGRC# 107870). RNAi lines carrying *UAS-dUCH-IR* fusion (GD#26468 or KK#103614) for knockdown *Drosophila Ubiquitin Carboxyl-terminal hydrolase* (*dUCH*, CG4265) were received from the Vienna *Drosophila* Resource Center (VDRC). dsRNAs produced from these RNAi lines are specific for *dUCH* (*CG4265*) mRNA among *UCH* genes (*CG4265*, *CG3431*, *CG1950*) in *Drosophila melanogaster* (Supplementary Fig. S4). RNAi line carrying *UAS-GFP-IR* for dsRNA control was obtained from BDSC (#9331). A number of GAL4 drivers were used to perform the targeted knockdown of *dUCH* in various tissues of *D. melanogaster*: *Act5C-GAL4* (BDSC#3954), *GMR-GAL4* (line #16)^53^, *MS1096-GAL4* (BDSC#8860), *pnr-GAL4* (BDSC#3039), *Ddc-GAL4* (BDSC#7009), *TH-GAL4* (BDSC#8848), and *D42-GAL4* (Bloomington #8816). All knockdown experiments using the GAL4/UAS system^54^ in the present study were performed at 28 °C in order to increase knockdown efficiency. Control flies were generated by crossing Canton-S with the respective GAL4 driver. Only male flies were used in experiments.

### Identification of the *Drosophila* ortholog of human and mouse UCHL1

The human UCHL1 protein sequence (hUCHL1, P09936) was retrieved from the UniProtKB/Swiss-Prot section of UniProtKB and used as a query sequence to run protein DELTA-BLAST^55^ in the non-redundant protein sequences database, which consists of GenBank CDS translations, PDB, SwissProt, PIR, and PRF (excluding environmental samples from WGS projects) with *D. melanogaster* (taxid:7227) as a targeted organism. The search result was manually analyzed to eliminate redundant sequences that caused the repetition of matched subject sequences in DELTA-BLAST results. These analyses were based on amino acid sequence comparisons, sequence features, and experimental data, which are provided in each subject sequence report. The conserved domains of the remaining sequences were identified by a CD search of the CDD v3.12 database in order to clarify whether these sequences contain the cysteine peptidase C12 domain of human UCHL1. Potential sequences were assembled to align with the human UCHL1 sequence and calculate identities using the Clustal Omega algorithm. The *Mus musculus* ortholog of human UCHL1 (mUCHL1, Q9R0P9) was also included in the alignment to observe the conservation of these sequences in three species. The alignment was visualized to overview identical positions and then analyzed (calculating conservation to show similarity based on the AMAS method of a multiple sequence alignment analysis^56^; adding sequence features such as active sites, important sites for hydrolytic activity, dimerization, and ligase activity, ubiquitin-binding sites, peptide-binding sites, and inhibitor-binding sites to observe the conservation of functional sites in these sequences) by Jalview 2.8.1^57^. Candidates for the ortholog of human UCHL1 were then reconfirmed by comparisons of its enzymatic activities published in previous studies on human UCHL1.

### Immunostaining, imaging, and DA neuron quantification

Immunohistochemistry was performed using a standard protocol^58^ with the following modifications. Larval and adult brains or eye imaginal discs were dissected in cold phosphate-buffered saline (PBS) and fixed in 4% paraformaldehyde at 25 °C for 15 min. After washing with 0.3% PBS-T (PBS containing 0.3% Triton-X100) twice, the brains were blocked in blocking solution (0.15% PBS-T containing 10% normal goat serum) at 25 °C for 20 min. Brains or eye imaginal discs were then incubated with the following primary antibodies diluted in blocking solution: rabbit anti-*Drosophila* Ubiquitin Carboxyl-terminal Hydrolase (anti-dUCH; 1:500)^59^ at 4 °C for 16 hrs or rabbit anti-Tyrosine Hydroxylase (anti-TH; 1:250; Millipore, AB152) at 4 °C for 20 hrs. After washing with 0.3% PBS-T, brains were incubated with secondary antibodies conjugated with Alexa 488 or FITC (1:500; Invitrogen) at 25 °C for 2 hrs, and then washed and mounted in Vectashield Mounting Medium (Vector Laboratories, Japan). Brains and eye imaginal discs were observed using Olympus Confocal FV10i FluoView System (Olympus, Japan). Brains were observed and scanned through the z-dimension to manifest the whole DA neuronal system and produce z-stack images. The number of DA neurons in each cluster was manually counted using the cell counter plugin of ImageJ (NIH, USA) based on the z-stack images produced.

### Western blot

Western blotting was performed using a standard protocol^58^ with the following modifications. Control (+; +; *Ddc-GAL4/*+) and knockdown (+; +; *Ddc-GAL4/UAS-dUCH-IR*) flies were frozen in liquid nitrogen and homogenized in lysis buffer. Homogenates were centrifuged and extracts (200 μg protein) were electrophoretically separated on SDS polyacrylamide gels containing 10% acrylamide, then transferred to polyvinylidene-difluoride membranes (BioRad, HCMC, Vietnam). The blotted membranes were blocked with Tris-buffered saline/0.05% Tween 20 containing 5% skim milk at 25 °C for 1 h, followed by an incubation with rabbit polyclonal anti-dUCH^59^ (1:1,000) or mouse monoclonal anti-α-tubulin IgG (1:5,000, Developmental Studies Hybridoma Bank, DSHB, Iowa, USA) at 4 °C for 16 h. After being washed, the membranes were incubated with horseradish peroxidase-conjugated secondary antibodies (GE Healthcare Bioscience, HCMC, Vietnam) at a 1:10,000 dilution at 25 °C for 1 h. Detection was performed with ECL Western blotting detection reagents (GE Healthcare Bioscience, HCMC, Vietnam) and images were quantified with a Lumivision ProHS II image analyzer (Aisin Seiki, Kariya, Japan).

### Crawling assay

The crawling assay was performed as described previously^43^ with the following modifications. A cohort of 10, 28, or 37 larvae in the third instar stage was randomly picked up from either the *dUCH* knockdown or control groups. These larvae were rinsed with PBS to discard food traces and treated with fluorescent light for 5 min. In the assay, each pair of the L3 larvae was placed on a 12 × 12 cm petri dish coated with 2% agar to continuously examine crawling ability at 28 °C. Larval movement was recorded using a Nikon Coolpix X100 digital camera for 60 sec at the highest resolution. The recorded videos were converted into 15 frames per second motion JPEG by the MOV to AVI converter (Pazera Jacek, Poland) and then analyzed by ImageJ (NIH, USA) with the wrMTrck plugin (developed by Dr. Jesper Søndergaard Pedersen, http://www.phage.dk/plugins/wrmtrck.html) to track larval movement and draw motion paths. Raw data were collected by Microsoft Excel 2010 (Microsoft, USA). The average velocity of each genotype was calculated, statistically analyzed, and graphed using GraphPad Prism 6.0 (GraphPad Software, USA). The experiment was conducted in five independent cohorts to confirm reproducibility.

### Startle-induced negative geotaxis assay (climbing assay)

The locomotor ability of adult flies was estimated using the startle-induced negative geotaxis assay or climbing activity^60^, which was then utilized as a method to evaluate locomotion defects caused by damage to neurons, specially DA neurons^19,47,61^. This assay was performed as described previously^62^ with the following modifications. Male flies from the desired genotypes were anesthetized with diethyl ether, grouped into cohorts of ten, and rested in separated food vials. Anesthetization was performed once prior to the assay and these flies were then transferred from food vials to climbing cylinders and back without anesthetizing in the entire experiment. Three independent cohorts were recruited from each genotype in the same experiment to perform repeated measures. Flies from a set of age-matched cohorts from the control and knockdown groups were separately placed into 100-ml polystyrene cylinders. The flies in these cylinders were tapped to the bottom and the movement of flies was recorded by the Nikon Coolpix X100 digital camera for 10 sec to produce a movie file. The number of flies that reached the 50-ml mark in 10 sec was manually counted based on the movie. Three trials were performed for a set of cohorts. The assay was performed every 5 days (from day 1 after eclosion to the day that none of the genotypes reached the 50-ml mark). The same group of flies was measured over time (from day 1 to 40). Raw data were collected and the percentage of flies that reached the 50-ml mark of each cohort was calculated by Microsoft Excel 2010 (Microsoft, USA). Data were then statistically analyzed by a repeated measures two-way ANOVA with Bonferroni’s post hoc test and graphed by GraphPad Prism 6.0 (GraphPad Software, USA). Experiments were repeated in triplicate to confirm reproducibility.

### Dopamine quantification

The dopamine quantification procedure was performed as described previously^47,63^ with the following modifications. Thirty fly heads were homogenized in 600 μl homogenization buffer (0.1 M perchloric acid/3% trichloro acid) on ice, sonicated 5 times for 30 sec each, and then placed on ice for 30 min. Debris was removed by centrifugation at 15,000 × *g* at 4 °C for 15 min. Fifty microliters of the supernatant was utilized for the HPLC analysis using Nanospace SI-2 (Shiseido, Japan) with running buffer containing 180 mM chloroacetic acid, 50 μM EDTA, 160 mM sodium hydroxide, and 8.5% acetonitrile. Samples were separated on a CapCell Pak C18 UG120 column (Shiseido, Japan) at a 0.5 ml/min flow rate. Dopamine was electrochemically detected by Electrochemical Detector 3005 (Shiseido, Japan). Dopamine (H8502, Sigma-Aldrich) was used to build the standard curve at 0.0025, 0.005, 0.01, 0.02, and 0.04 μM. Differences in the dopamine levels of the examined samples were statistically analyzed using an ordinary two-way ANOVA with Tukey’s multiple comparisons test and graphed by GraphPad Prism 6.0 (GraphPad Software, USA).

## Electronic supplementary material

Supplementary data
