# Supplementary material for: Drosophila Ubiquitin C-Terminal Hydrolase Knockdown Model of Parkinson’s Disease
Source: Sci Rep. 2018 Mar 13;8:4468. doi: 10.1038/s41598-018-22804-w (PMC5849740; doi:10.1038/s41598-018-22804-w)
Supplement: Supplementary file 1 — Supplementary data [file 41598_2018_22804_MOESM1_ESM.pdf]

Supplementary Information (SI)

Drosophila Ubiquitin C-Terminal Hydrolase Knockdown Model of Parkinson's Disease

Hiep H. Tran[1,+], Suong N. A. Dang[1,3,+], Thanh T. Nguyen[1], Anh M. Huynh[1], Linh. M. Dao[1], Kaeko Kamei[2], Masamitsu Yamaguchi[3,4], Thao T. P. Dang[1,*]

[1]Department of Molecular and Environmental Biotechnology, Faculty of Biology and Biotechnology, University of Science, Vietnam National University 70000, Vietnam.

[2]Department of Biomolecular Engineering, Kyoto Institute of Technology, Kyoto 606-8585, Japan

[3]Department of Applied Biology, Kyoto Institute of Technology, Kyoto 606-8585, Japan

[4]The Center for Advanced Insect Research Promotion, Kyoto Institute of Technology, Kyoto 606-8585, Japan

[+]These authors contributed equally to this work.

[*]Corresponding author: Thao T.P. Dang (dtpthao@hcmus.edu.vn)

Supplementary Table S1. Amino acid sequence identities between **(A)** human UCHL1 and *Drosophila melanogaster* UCH family proteins, and **(B)** dUCH and other *Drosophila melanogaster* UCH family proteins. Sequence identities indicated that CG3431 and CG1950 are not related to human UCHL1 and dUCH (CG4265).

**(A)**	**dUCH** (CG4265, P35122)	**dUCHL5** (CG3431, Q9XZ61)	**dUCHL5R** (CG1950, Q9VYQ3)
**hUCHL1** (P09936)	43.67%	14.07%	12.25%

**(B)**	**dUCHL5** (CG3431, Q9XZ61)	**dUCHL5R** (CG1950, Q9VYQ3)
**dUCH** (CG4265, P35122)	16.72%	17.05%

CG numbers: Flybase gene identification numbers, P or Q numbers: Uniprot protein identification numbers.

Supplementary Table S2. Phenotypic aberration in heterozygous *dUCH* knockdown flies. *Act5c*, *Actin 5C*; *GMR*, *Glass Multimer Reporter*; MF, Morphogenetic Furrow; *MS1096*, *MS1096-GAL4* is a GAL4 P-element insertion at the *Beadex* locus; *pnr*, *pannier*; *Ddc*, *Dopa decarboxylase*; *D42*, motor neuron-specific promoter; *TH*, *Tyrosine Hydroxylase*.

**GAL4 driver**	**Target cell/ tissue**	**Phenotype description**
Act5C	Ubiquitous expression	Pupal lethality
GMR	All cells posterior to MF of the third instar larval eye imaginal disc	Rough eye
MS1096	Entire wing pouch and some regions of the notum of the third instar larval wing imaginal disc	Abnormal vein pattern
pnr	Most proximal notum of the third instar wing imaginal disc	Abnormal bristle and hair patterns
Ddc	Dopaminergic and serotonergic neurons	Progressive locomotor deficit
D42	Motor neurons	Abnormal morphology of synapse at neuromuscular junction (Supplementary Figure S5)
TH	Dopaminergic neurons	Locomotor dysfunction and deficit

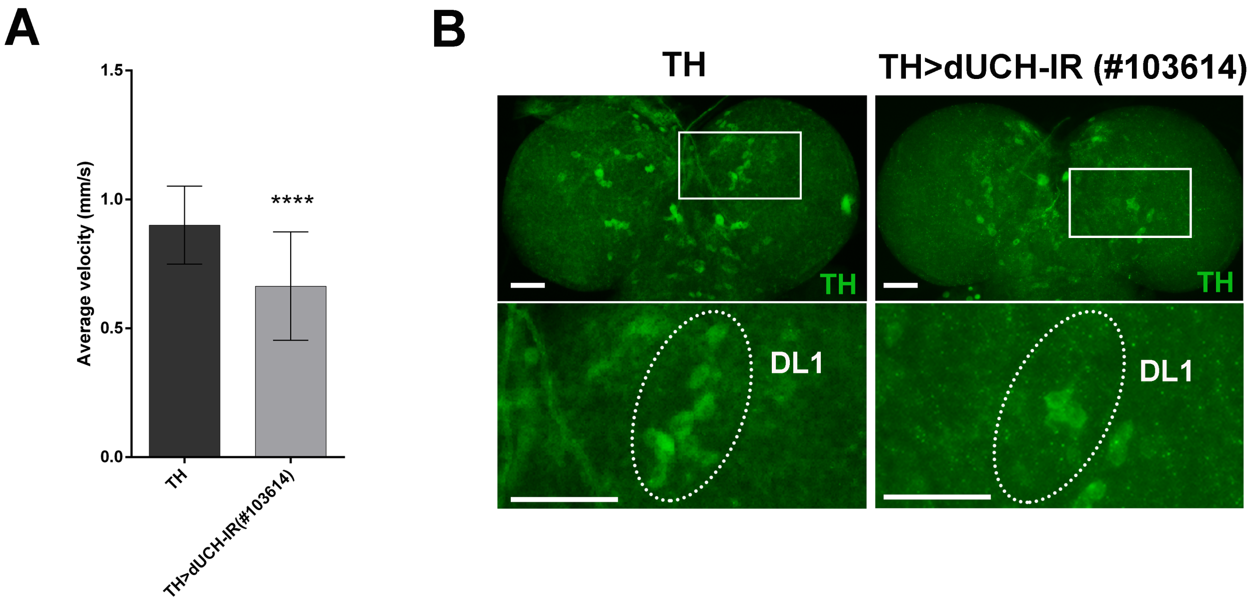

Supplementary Figure S1 (related to Fig.3 and Fig.4). Knockdown *dUCH* in DA neurons by a second RNAi construct (VDRC KK stock #103614) also showed the same phenotypes as in the VDRC GD stock #26468. **(A)** The #103614 *dUCH* knockdown larvae (TH>dUCH-IR#103614) also showed reduction in crawling velocity when compared to driver control (TH). **(B)** Loss of DL1 DA neurons also occurred in *dUCH* knockdown #103614 (TH>dUCH-IR#103614) comparing to driver control (TH). Scale bars indicate 50μm.

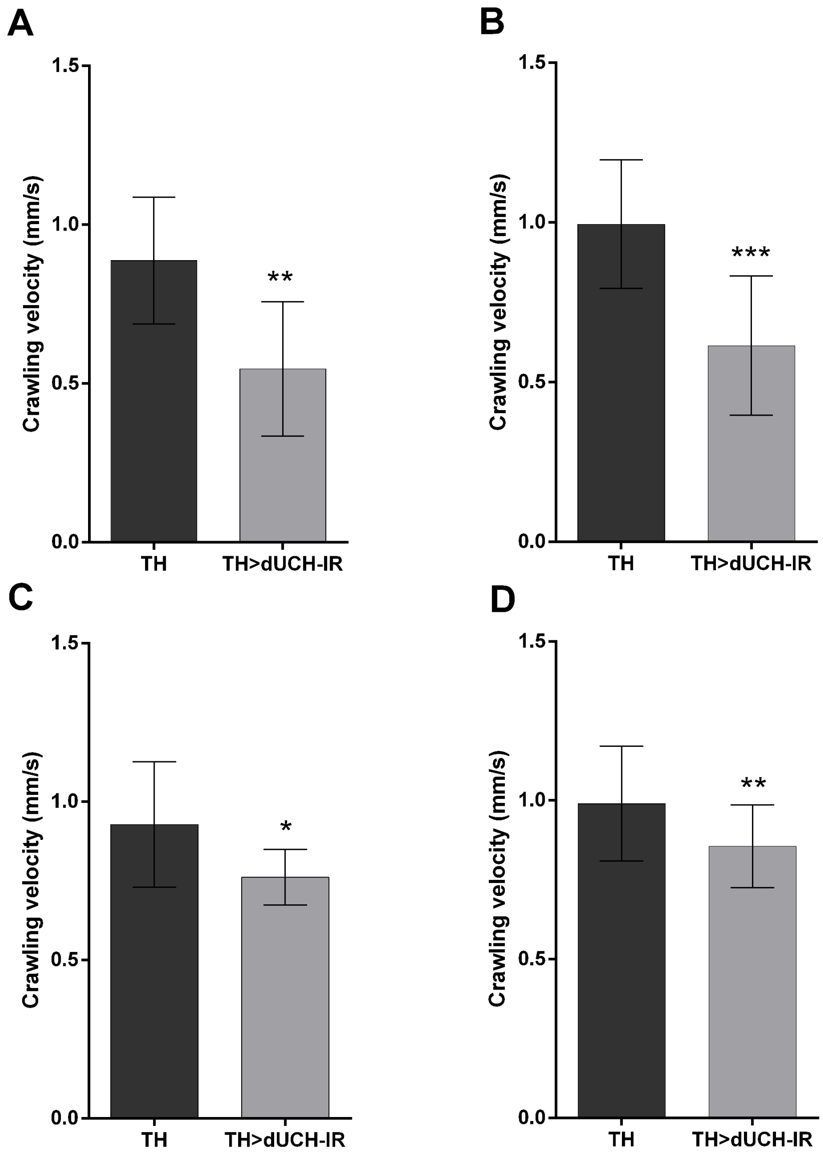

Supplementary Figure S2 (relate to Fig. 3). **(A-D)** Crawling velocity of driver control (TH) and *dUCH* knockdown larvae (TH>dUCH-IR) from four independent cohorts showed the reproducibility of results in Fig. 3B, unpaired Student's t-test with Welch's correction (A, n=10; B, n=10; C, n= 10, D, n=28), * $p< 0.05$, ** $p< 0.01$, *** $p< 0.001$, data are presented as the mean ± SD.

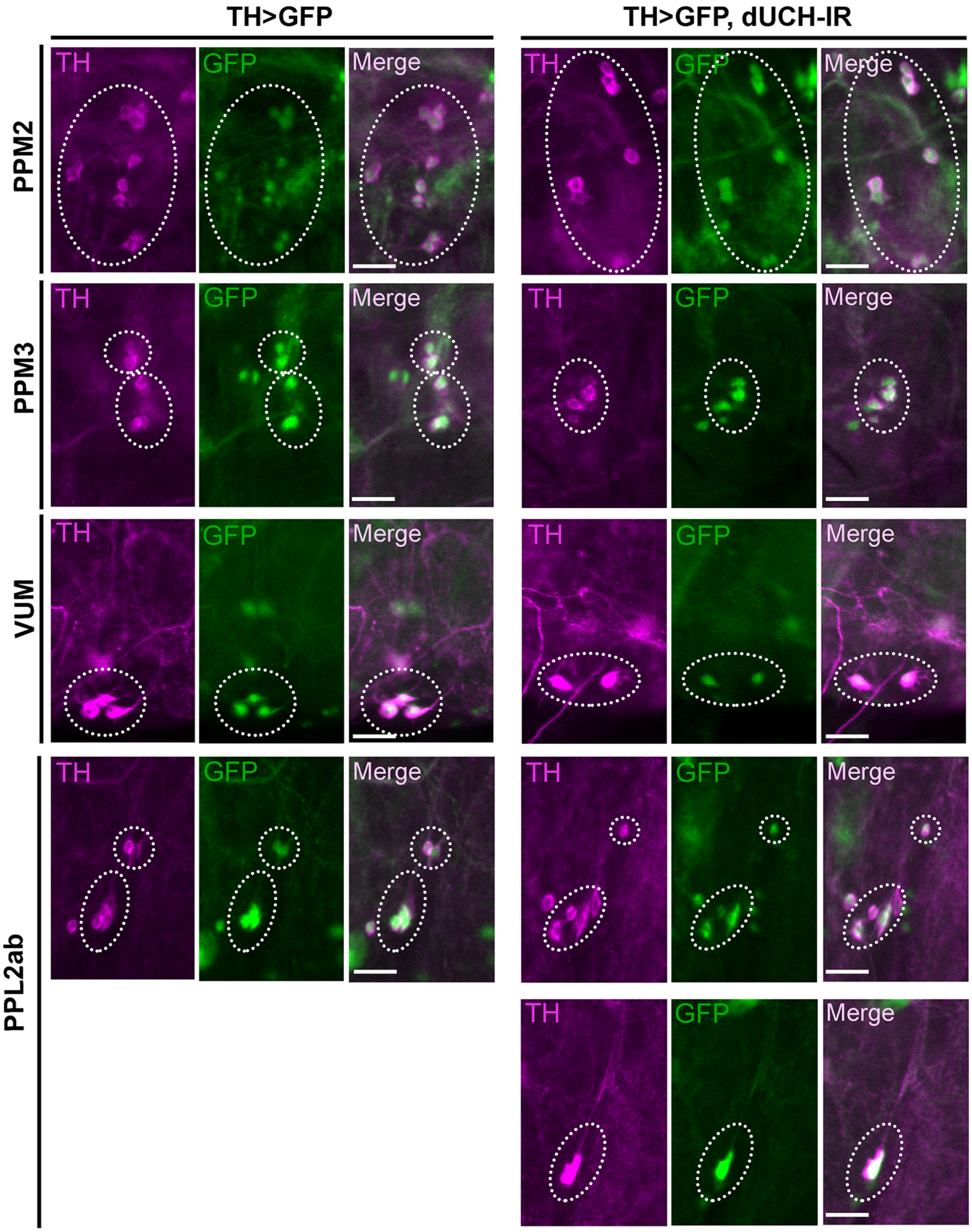

Supplementary Figure S3 (related to Fig.5). Loss of TH signals in *dUCH* knockdown adult brains correlated with loss of DA neurons. **(TH>GFP, dUCH-IR)** Flies with *dUCH* knockdown and GFP ectopic expression driven by TH-GAL4 (+*; UAS-GFP.nls/*+*; TH-GAL4/UAS-dUCH-IR*) showed DA neuron degeneration in examined clusters (PPM2, PPM3, VUM, and PPL2ab) comparing to control flies **(TH>GFP)** which expressed GFP under *TH-GAL4* driver (+*; UAS-GFP.nls/*+*; TH-GAL4/*+). DA neurons were detected by nuclear GFP (green) and immunostaining with anti-TH antibody (magenta). Scale bars indicate 20μm.

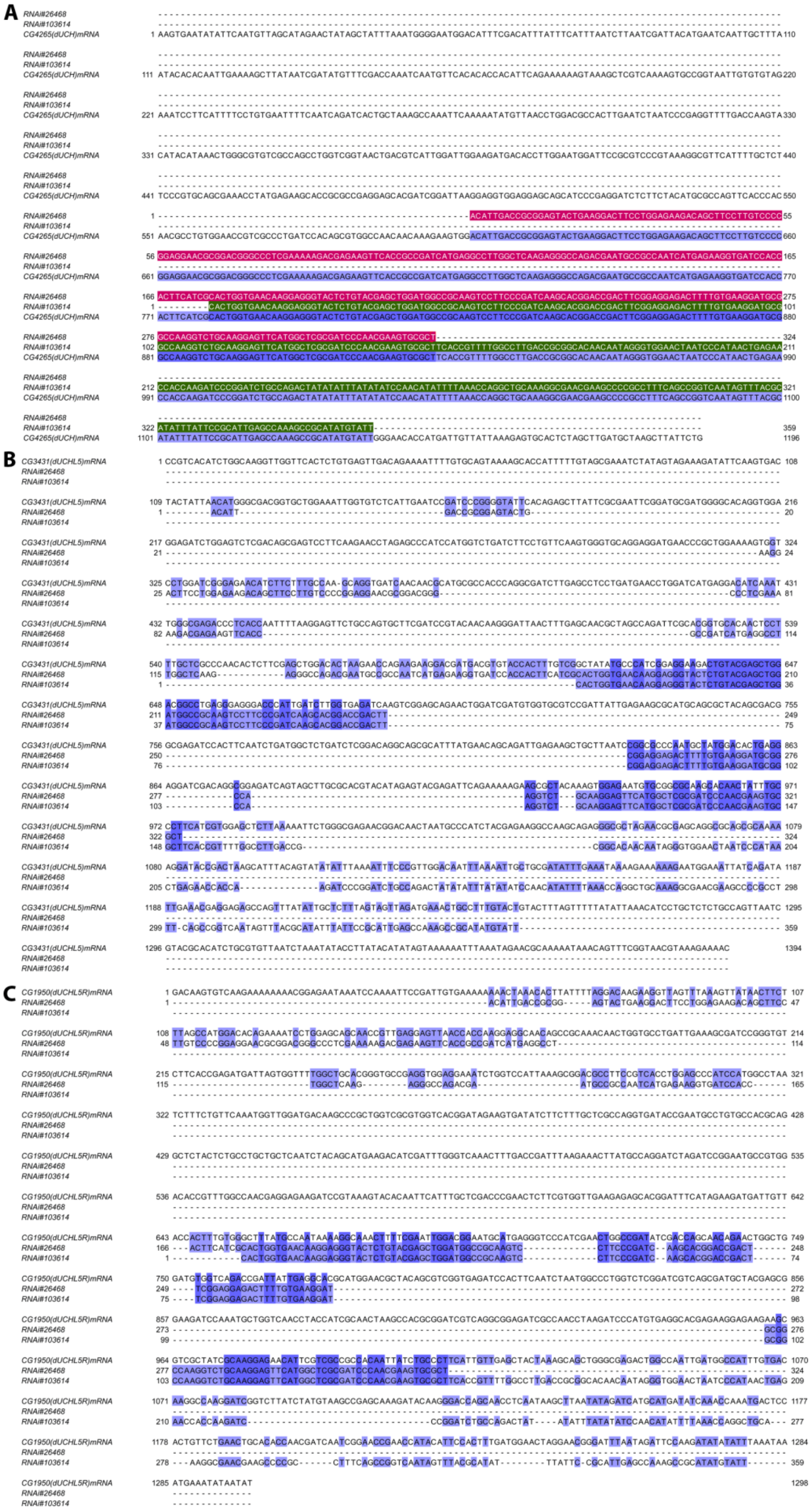

Supplementary Figure S4. Complementary regions of RNAi constructs (VDRC#26468 and VDRC#103614) and CG4265

(*dUCH*) mRNA **(A)**, CG3431 (*dUCHL5*) mRNA **(B)**, and CG1950 (*dUCHL5R*) **(C)** show specificity of these RNAi constructs to CG4265.

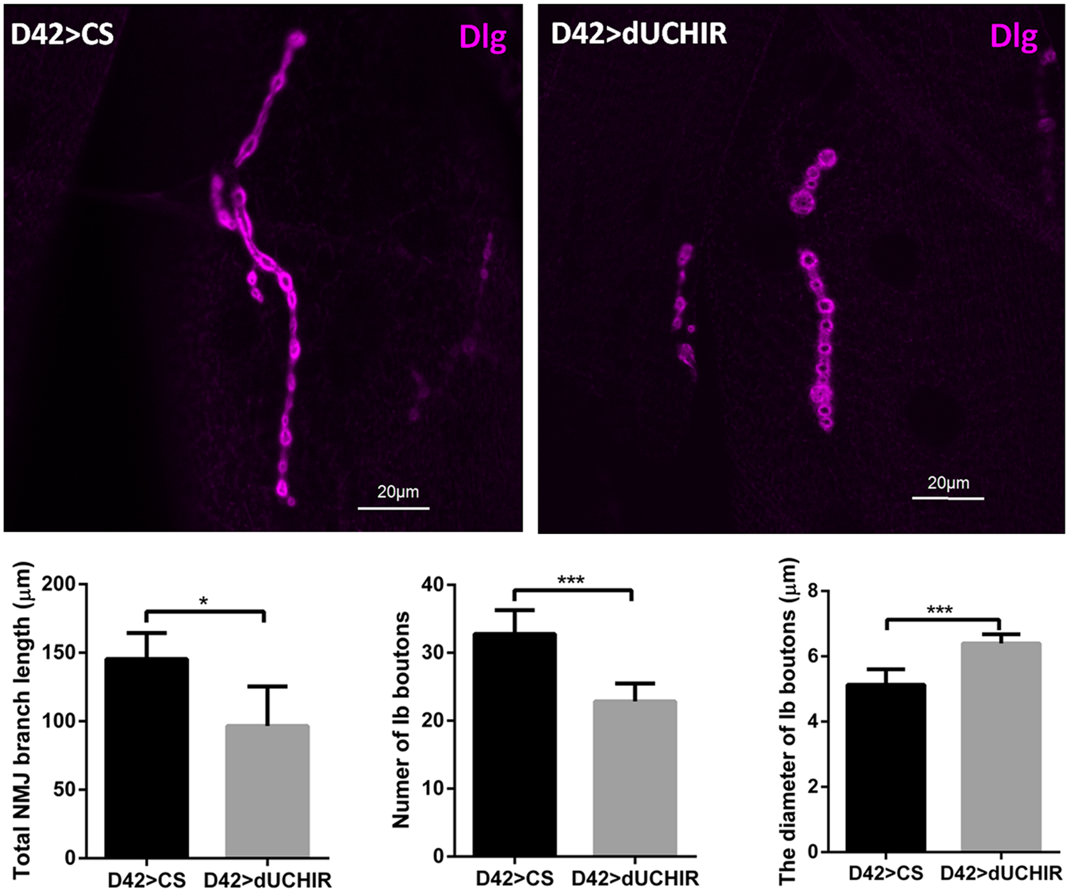

Supplementary Figure S5. Knockdown of *dUCH* in third instar larval motor neurons. Knockdown of *dUCH* in motor neurons by using *D42-GAL4* driver causes abnormal morphology of motor neuron presynaptic and postsynaptic terminals at neuromuscular junctions (NMJ) in muscle 4 of third instar larvae. NMJs were stained with anti-Discs large (Dlg) IgG (red). Scale bars indicate 20 μm. The quantified data shows the decrease in the NMJ branch lengths and number of Ib boutons, and the increase in the size of Ib boutons in *dUCH* knockdown larvae D42>dUCHIR (+*; +; D42-GAL4/UAS-dUCH-IR*) (VDRC #26468) in the comparison with that of control larvae D42>CS (+*; +; D42-GAL4/*+), (unpaired Student's *t*-test, *p<0.05 and *** p<0.001, n=5). These results indicated that dUCH plays an essential role in synapse structure formation at NMJs.
